# Update on Osteoporosis

**DOI:** 10.3390/medicina61020275

**Published:** 2025-02-06

**Authors:** Tommaso Greco, Carlo Perisano

**Affiliations:** 1 Orthopedics and Trauma Surgery Unit, Department of Aging, Orthopedic and Rheumatologic Sciences, Fondazione Policlinico Universitario Agostino Gemelli IRCCS, 00168 Rome, Italy; carlo.perisano@policlinicogemelli.it; 2 Department of Life Sciences, Health, and Health Professions, Link Campus University, 00165 Rome, Italy

Osteoporosis remains a major public health concern, significantly impacting the quality of life of the elderly and those with conditions that are associated with reduced bone density [1]. In this first edition of the Special Issue of *Medicina* titled “Update on Osteoporosis”, scholars from around the world were invited to provide innovative contributions. The included articles provide an overview of the most recent developments in the management and understanding of osteoporosis, with a focus on gender and population differences, as well as innovative therapeutic strategies. To achieve the most up-to-date overview possible, the impact on osteoporosis of the recent COVID-19 pandemic was also considered [2]. 

Kim and Kong explored differences in femoral curvature in the Korean population, showing significant variations related to age and gender [3]. Their research underscores the importance of considering population-specific anatomical features in clinical approaches, particularly for surgical planning and fracture prevention. This study emphasizes and aims to direct attention to ethnic and gender differences in bone structure, a crucial aspect in personalizing treatment.

Kwon et al. compared the impact of Teriparatide and Denosumab on osteoporotic vertebral compression fractures, providing a detailed analysis of the clinical and radiographic outcomes [4]. This study highlights the importance of targeted pharmacological management to improve fracture healing and prevent recurrence, helping to outline more effective treatment strategies for patients with advanced osteoporosis.

Also analyzing vertebral fractures, Yoshimi et al. focused their attention on the challenge of pain assessment in hospitalized patients, especially those with cognitive impairment [5]. The introduction of observational assessment tools offers new possibilities for pain monitoring and management in this vulnerable population, improving clinical practices and, potentially, long-term outcomes.

Nozaki et al. showed that the COVID-19 pandemic did not reduce the incidence of hip fractures among the elderly in Niigata Prefecture [6]. This study highlights the resilience of public health dynamics and the importance of maintaining epidemiological surveillance, even during global crises, to ensure an adequate and timely health response.

Osteoporosis is a disorder that affects the female sex most frequently, although it can impact the male sex with effects that are equally devastating. Nishimoto et al. analyzed the incidence of osteoporosis in men undergoing androgen deprivation therapy for prostate cancer [7]. The authors highlight the need to actively monitor and treat bone density loss in this population and propose specific preventive and therapeutic interventions.

This Special Issue points out the complexities of osteoporosis and the need for individualized approaches to managing the disease, helping not only to broaden our understanding of the disease but also proposing practical solutions to address the associated clinical challenges.

Looking ahead, it is essential to promote further large-scale longitudinal studies to better understand the genetic and environmental variations that influence the osteoporosis risk in different populations. In addition, the development of new biomarkers and advanced imaging technologies could improve early diagnosis and monitoring of disease progression. Innovation in drug treatments, with a focus on combination therapies and personalization based on a patient’s genetic profile, could revolutionize the way in which we manage this disease.
